# A Comparative Study Between Posterolateral Lumbar Fusion and Transforaminal Lumbar Interbody Fusion for Degenerative Lumbar Spondylolisthesis

**DOI:** 10.7759/cureus.76022

**Published:** 2024-12-19

**Authors:** Basanth Reddy A, Nagakumar J S., Manoj K Ramachandraiah

**Affiliations:** 1 Department of Orthopedics, Sri Devaraj Urs Academy of Higher Education and Research, Kolar, IND

**Keywords:** clinical outcomes, degenerative spondylolisthesis, posterolateral lumbar fusion, radiographic parameters, transforaminal lumbar interbody fusion, visual analogue scale

## Abstract

Introduction

Degenerative spondylolisthesis (DS) is a degenerative condition characterized by subluxation of one vertebral body anterior to the adjacent inferior vertebral body with an intact pars. Conservative treatment approaches, such as steroid injections and physical therapy, may work well at first, but in resistant situations, surgery is frequently necessary. Posterolateral lumbar fusion (PLF) has been widely used, but transforaminal lumbar interbody fusion (TLIF) offers theoretical advantages such as improved alignment and enhanced fusion rates.

Methods

This retrospective study examined patients with DS who underwent PLF or TLIF at R.L. Jalappa Hospital and Research Centre, Kolar, Karnataka, India, between January 2020 and January 2024. The inclusion criteria were planned one- or two-level fusion DS resistant to conservative treatment and at least one year of follow-up. Patients with prior lumbar fusion or uncontrolled comorbidities were not included. Details were taken from medical records and Picture Archiving and Communication System (PACS), and SPSS Version 22 was used for analysis. Continuous variables were compared using t-tests or Mann-Whitney U tests, and categorical variables were compared using chi-square tests. The non-inferiority of TLIF to PLF was assessed with predefined margins. The study included 56 patients (28 in each group), with outcomes measured via visual analogue scale (VAS), 12-Item Short Form Survey (SF-12) scores, and radiological outcomes. The threshold for clinical significance was p < 0.05.

Results

This research compared TLIF and PLF in a total of 56 DS patients. Demographic data, operative details, and pre-surgical parameters showed no significant differences. Clinical outcomes revealed comparable improvements in scores for leg and back pain (p > 0.05). SF-12 Physical Component Summary (PCS) scores were significantly higher post-surgery in the TLIF group (42.4 ± 5.1) compared to the PLF group (37.9 ± 4.5) (p = 0.01). Radiologically, the slippage was significantly lower in the TLIF cohort (2.1 ± 1.02) compared to the PLF cohort (3.1 ± 1.9) (p = 0.01). Both anterior and posterior disc heights were substantially increased in the TLIF group (11.3 ± 1.6 vs. 9.3 ± 1.5 in PLF unit, p < 0.01) compared to the PLF group (1.7 ± 0.9 vs. 0.8 ± 0.4, p < 0.01). There were no discernible variations in lumbar lordosis, sacral slope, or changes in the tilt of the pelvis. TLIF demonstrated better radiographic improvements but had clinical outcomes similar to those of PLF.

Conclusion

TLIF demonstrated superior radiographic improvements, particularly in disc height and slippage reduction, while clinical outcomes were comparable between TLIF and PLF, suggesting that both are effective options for managing DS.

## Introduction

Degenerative alterations in the vertebrae and intervertebral discs cause degenerative spondylolisthesis (DS), which is the forward movement of a vertebral body relative to the vertebra below.

Spondylolisthesis is frequently observed in middle-aged and older individuals with chronic back pain, yet there is limited research on its comprehensive radiological characteristics. The pathophysiology of DS remains a topic of debate, with some theories suggesting that degeneration of the facet joints and intervertebral discs contributes to segmental instability, leading to vertebral displacement over time [1].

This disorder frequently results in spinal stenosis, facet joint arthritis, instability, or nerve root compression, which can cause symptoms including neurogenic claudication, radiculopathy, or lower back pain [2]. Conservative management is initially the first line of treatment, but if there is no improvement in symptoms, surgical management is indicated.

Surgical approaches for DS generally include decompression alone or decompression with fusion to stabilize the spine. Cloward originally defined PLF in 1940, and after Lin suggested some changes, it was widely adopted [3].

Posterolateral lumbar fusion (PLF) surgery is performed commonly, but transforaminal lumbar interbody fusion (TLIF) has the advantages of better alignment and early fusion rates according to theory [4-6]. Despite these theoretical benefits, clinical studies comparing PLF and TLIF have reported inconsistent results [7,8]. The purpose of this study is to fill this information gap by comparing radiographic and clinical outcomes of PLF and TLIF for DS in a rural Indian population.

## Materials and methods

This retrospective cohort study design is for evaluating the clinical results and radiographic outcomes of two common surgical methods for DS: PLF and TLIF. The patients included in this analysis were selected based on their medical records, which were reviewed retrospectively. Information was retrieved from the hospital’s medical files of patients who underwent either PLF or TLIF for DS between January 2020 and January 2024. The study only included patients who had been followed up with for at least a year to ensure that outcomes could be properly assessed over a sufficient time frame for both clinical and radiological changes. All the outcomes were measured at the end of one year of the final follow-up period.

Cases of DS surgically managed with PLF and TLIF at R.L. Jalappa Hospital and Research Centre were chosen retrospectively from January 2020 to January 2024. Patient data were retrieved from hospital records, including clinical notes, operative details, and radiographic data stored in the Picture Archiving and Communication System (PACS).

For this retrospective cohort study comparing TLIF and PLF outcomes in DS, a sample size calculation was performed for non-inferiority testing. Based on Takahito Fujimori et al.'s data [2], with a standard deviation of 9 for the 12-Item Short Form Survey (SF-12) score, a non-inferiority margin of 5, with significance (α) of 0.05, and power (1-β) of 0.8, the needed sample size was 28 patients per cohort, resulting in a total of 56 patients. This ensures sufficient power to detect meaningful changes between the groups.

Inclusion and exclusion criteria

The inclusion criteria included clinicoradiologically diagnosed DS cases resistant to conventional treatment, those who underwent one or two-level instrumented fusion, and availability of medical and radiological data with follow-up for a minimum of a year. The exclusion criteria included a history of previous lumbar fusion surgery and patients with uncontrolled comorbidities.

Study objectives

The visual analog scale (VAS) for pain and the SF-12 score, which measures life quality, were the main clinical outcome measures. The VAS score was used to gauge the patient's level of pain, and the SF-12 score was used to gauge their functional status and general well-being, with an emphasis on the physical and mental health domains.

Radiographic parameters were analysed for vertebral slippage, disc height, local disc and lumbar lordosis, and pelvic parameters such as sacral slope and pelvic tilt on pre- and post-operative X-rays. All the outcomes were measured at the end of one year of the final follow-up period.

Statistical analysis

SPSS Version 22 (IBM Corp., Armonk, NY) was used for processing the data. The mean (SD) was used for summarizing the continuous variables, and t-tests or Mann-Whitney U tests were used for non-normally distributed characteristics. We used the chi-square test for assessing categorical variables. The non-inferiority of TLIF compared to PLF was assessed using predefined margins. The threshold for statistical significance was p<0.05.

Ethical consideration

This study was approved by the central ethics committee of Sri Devaraj Urs Academy of Higher Education and Research, with reference number SDUAHER/KLR/R&D/CEC/S/PG/74/2024-2025. All patient data were anonymized, and the Declaration of Helsinki's guiding principles were followed in this investigation.

## Results

A total of 56 participants were included in this study. Demographic and operative details of our study groups are listed in Table 1. The TLIF unit comprised 17 (60.7%) males and 11 (39.2%) females, with an average age of 53.9 ± 1.00 years, while the PLF unit included 15 (53.5%) males and 13 (46.4%) females, with an average age of 56.4 ± 0.99 years. There was not a statistically significant distinction in the age patterns of the two groups (p = 0.09). Diabetes was present in 28.6% of the TLIF unit and 21.4% of the PLF unit; these differences were not statistically significant (p = 0.54). In both units, the number of levels involved, that is, one or two levels in surgery, was similar, with 20/8 in the PLF unit and 22/6 in the TLIF unit (p = 0.54). Additionally, there was little change between the groups in terms of fusion levels and bone graft types (local vs. iliac crest) (p > 0.05).

**Table 1:** Demographic and operative features of TLIF and PLF units (N= 56) The data are presented as n% or mean ± SD. A p-value of ≤0.05 is considered significant The p-value was not calculated for the fusion level PLF, posterolateral lumbar fusion; TLIF, transforaminal lumbar interbody fusion

Variable	TLIF, n = 28 (%)	PLF, n = 28 (%)	p-Value
Age	53.9±1.00	56.4±0.99	0.09
Gender			
Male	17 (60.7%)	15 (53.5%)	0.59
Female	11(39.2%)	13 (46.4%)	
Diabetes (%)	8 (28.6%)	6 (21.4%)	0.54
Number of levels (1/2)			
1	22 (78.5%)	20 (71.4%)	0.54
2	6 (21.4%)	8 (28.5%)	
Fusion level			
L3-L4	5 (17.9%)	4 (14.3%)	-
L4-L5	19 (67.9%)	21 (75.0%)	
L5-S1	4 (14.3%)	3 (10.7%)	
Bone graft			
Iliac crest	24 (85.7%)	20 (71.4%)	0.19
Local	4 (14.3%)	8 (28.5%)	

Comparison of clinical outcomes

The VAS for low back pain, right leg, and left leg were compared before and after surgery in both groups (Table 2). Pre-surgery, both groups had similar VAS scores for low back ache, right leg, and left leg (all p = 1). Post-surgery, the VAS for low back ache was 2.7 ± 0.59 in the TLIF unit and 2.9 ± 0.71 in the PLF unit (p = 0.28). The change in VAS for low back pain was -4.75 ± 1.04 in the TLIF unit and -4.53 ± 1.07 in the PLF unit, which was not statistically significant (p = 0.42). Similarly, no significant variation was found for VAS scores of right and left leg pain pre- and post-surgery or the variation in the groups' scores (all p > 0.05). The SF-12 Physical Component Summary (PCS) scores, as shown in Table 2 before surgery, were similar in both groups (30.1 ± 1.7 in the TLIF unit and 28.9 ± 4.0 in the PLF unit, p = 0.53). Post-surgery, the TLIF group had a significantly higher PCS score (42.4 ± 5.1) compared to the PLF group (37.9 ± 4.5) (p = 0.01). The improvement in the TLIF group (12.4 ± 4.1) was greater than the improvement in the PLF group (9.6 ± 2.7); however, this disparity was not statistically significant (p = 0.07). SF-12 Mental Component Summary (MCS) scores, as shown in Table 2, were similar in both groups before and after surgery (pre-surgery: TLIF 39.4 ± 7.3 vs. PLF 39.5 ± 6.9, p = 0.98; post-surgery: TLIF 51.2 ± 6.7 vs. PLF 51.5 ± 6.8, p = 0.79). There was no discernible variation in the group changes in MCS scores (TLIF 11.8 ± 4.2 vs. PLF 12.1 ± 4.9, p = 0.81).

**Table 2:** Comparison of clinical results between the TLIF and PLF groups (N= 56) The data are presented as n%, mean ± SD *A p-value of ≤0.05 is considered significant PLF, posterolateral lumbar fusion; SF-12 PCS, 12-Item Short Form Survey Physical Component Summary; SF-12 MCS, 12-Item Short Form Survey Mental Component Summary; TLIF, transforaminal lumbar interbody fusion; VAS, visual analog scale

Variable	Group TLIF (n = 28)	Group PLF (n = 28)	p-Value
	Mean ± SD	Mean ± SD	
VAS low back pain			
Pre-surgery	7.46 ± 0.69	7.46 ± 0.69	1
Post-surgery	2.7 ± 0.59	2.9 ± 0.71	0.28
Change	-4.75 ±1.04	-4.53 ± 1.07	0.42
VAS right leg			
Pre-surgery	7.93 ± 0.77	7.93 ± 0.77	1
Post-surgery	2.04 ± 0.58	2.02 ± 0.59	0.17
Change	-5.9 ± 0.74	-5.7 ± 0.94	0.5
VAS left leg			
Pre-surgery	7.94 ± 0.77	7.93 ± 0.77	1
Post-surgery	2.04 ± 0.58	2.2 ± 0.72	0.4
Change	-5.9 ± 0.74	-5.8 ± 0.84	0.6
SF-12 PCS			
Pre-surgery	30.1 ± 1.7	28.9 ± 4	0.53
Post-surgery	42.4 ± 5.1	37.9 ± 4.5	0.01*
Change	12.4 ± 4.1	9.6 ± 2.7	0.07
SF-12 MCS			
Pre-surgery	39.4 ± 7.3	39.5 ± 6.9	0.98
Post-surgery	51.2 ± 6.7	51.5 ± 6.8	0.79
Change	11.8 ± 4.2	12.1 ± 4.9	0.81

Comparison of radiographic parameters

The radiographic parameters such as slippage, disc height, local disc and lumbar lordosis, and pelvic parameters were compared before and after surgery in both groups (Table 3).

**Table 3:** Correlation of radiographic parameters between the TLIF and PLF groups (N= 56) The data are presented as n% or mean ± SD *A p-value of ≤0.05 is considered significant PLF, posterolateral lumbar fusion; TLIF, transforaminal lumbar interbody fusion

Variable	Group TLIF (n = 28)	Group PLF (n = 28)	p-Value
	Mean ± SD	Mean ± SD	
Slippage			
Pre-surgery	5.0 ± 1.8	5.5 ± 1.9	0.36
Post-surgery	2.1 ± 1.02	3.1 ± 1.9	0.01*
Change	-2.9 ± 0.98	-2.4 ± 0.84	0.03*
Local disc lordosis			
Pre-surgery	6.1 ± 2.5	4.7 ± 2.0	0.03*
Post-surgery	7.4 ± 2	5.5 ± 1.7	<0.01*
Change	1.3 ± 1.2	0.79 ± 0.62	0.06
Anterior disc height			
Pre-surgery	9.6 ± 1.8	8.3 ± 1.4	<0.01*
Post-surgery	11.3 ± 1.6	9.3 ± 1.5	<0.01*
Change	1.7 ± 0.84	1 ± 0.7	<0.01*
Posterior disc height			
Pre-surgery	6.1 ± 0.9	5.2 ± 0.8	<0.01*
Post-surgery	7.8 ± 0.53	6 ± 0.7	<0.01*
Change	1.7 ± 0.9	0.8 ± 0.4	<0.01*
Lumbar lordosis			
Pre-surgery	49 ± 9.6	49.4 ± 9.5	0.89
Post-surgery	55.5 ± 8.4	55.1 ± 8.2	0.79
Change	6.5 ± 3.4	5.7 ± 2.9	0.44
Sacral slope			
Pre-surgery	32.4 ± 5.9	33.7 ± 3.9	0.34
Post-surgery	36.9 ± 5.1	37.8 ± 3.6	0.69
Change	4.5 ± 2.1	4.0 ± 1.8	0.34
Pelvic tilt			
Pre-surgery	18.2 ± 4.3	17.9 ± 3.4	0.73
Post-surgery	21.6 ± 5.3	21.1 ± 4.5	0.64
Change	3.4 ± 2.9	3.4 ± 2.9	0.73

Pre-surgery, the disc slippage was similar between the groups (5.0 ± 1.8 in TLIF vs. 5.5 ± 1.9 in PLF, p = 0.36). Post-surgery, however, the slippage was significantly lower in the TLIF cohort (2.1 ± 1.02) compared to the PLF cohort (3.1 ± 1.9) (p = 0.01). Additionally, the TLIF unit experienced a greater decrease in slippage (-2.9 ± 0.98 vs. -2.4 ± 0.84, p = 0.03).

Pre-surgery, the local disc lordosis was higher in the TLIF cohort (6.1 ± 2.5) as opposed to the PLF group (4.7 ± 2.0) (p = 0.03). Post-surgery, the TLIF cohort showed a significantly greater improvement in local disc lordosis (7.4 ± 2.0) in contrast to the PLF group (5.5 ± 1.7) (p < 0.01). Furthermore, the TLIF group showed a larger shift in local disc lordosis (1.3 ± 1.2) than the PLF group (0.79 ± 0.62); however, the change was not statistically significant (p = 0.06).

Both anterior and posterior disc heights demonstrated great progress in post-surgery in both categories. Pre-surgery, the TLIF group had a significantly higher anterior disc height (9.6 ± 1.8) in comparison with the PLF unit (8.3 ± 1.4) (p < 0.01). Similarly, the TLIF group's anterior disc height increased more after surgery (11.3 ± 1.6 vs. 9.3 ± 1.5 in PLF unit, p < 0.01). The posterior disc height was also significantly greater in TLIF unit pre- and post-surgery (p < 0.01), with a more substantial change in the TLIF group (1.7 ± 0.9 vs. 0.8 ± 0.4, p < 0.01).

Lumbar lordosis, sacral slope, and pelvic tilt did not demonstrate any significant variation between the groups before and after surgery. Additionally, the changes in these characteristics were not different between both groups (all p > 0.05).

## Discussion

The objective of this study was to compare the clinical and radiographic results of posterior lumbar fusion (PLF) with TLIF among patients having DS. The analysis covered demographic details, clinical outcomes, and radiographic parameters to determine the effectiveness of the two surgical techniques.

No discernible differences existed between the two groups' demographic information, such as age, gender, comorbidities, or other preoperative features. In the TLIF group, there were 17 males and 11 females, with a mean age of 53.9 ± 1.00 years, while the PLF unit had 15 males and 13 females, with a mean age of 56.4 ± 0.99 years. These results are somewhat comparable to an Indian study conducted by Yadav et al. [9], in which the mean age was reported as 47.62 ± 14.7 years in the PLF group and 48 ± 8.5 years in the TLIF group. In our study, the p-value for age was 0.09, indicating no discernable distinction between the two units. In our study, the prevalence of diabetes was 28.6% in the TLIF unit and 21.4% in the PLF unit, with a p-value of 0.54, showing no statistical difference. Another systematic review and meta-analysis conducted by Zhang et al. [10] reported that the demographic characteristics of both groups were not comparable, with no significant distinctions in age group, gender, or comorbidities.

TLIF and PLF showed similar clinical or survival outcomes and rates of complication in treating degenerative lumbar disorders [11,12]. In our current study, the number of levels associated with fusion was also consistent among the groups, with 22 patients in the TLIF group having a single-level fusion and six having two-level fusions, compared to 20 single-level and 8 two-level fusions in the PLF group. The p-value for the number of levels involved was 0.54, indicating no significant difference. Several international studies reported similar results with the number of levels involved in fusion, which was similar between the TLIF and PLF groups, with no significant difference in reoperation rates or results reported by patients [11,13].

The effectiveness of iliac crest bone graft (ICBG) versus local bone graft in lumbar spinal fusion surgeries has been examined in several studies. In a study conducted by Ohtori et al. in single-level posterolateral fusion, local bone graft achieved similar success in fusion rates and clinical outcomes as ICBG, with fewer complications and shorter surgical time [14]. For posterior lumbar interbody fusion (PLIF), two studies found not much difference in fusion rates between local bone and ICBG groups, with final fusion rates above 94% for both graft types [15].

In our study regarding the bone graft source, 24 patients in the TLIF group received ICBG, while four received the local bone graft. Among the PLF group, 20 received an ICBG and eight received a local bone graft. The p-value for this comparison was 0.19, indicating that there was no discernible variation in the group's choices of grafts.

In our study, both groups showed substantial improvement in pain levels. However, no statistically discernable variations were observed in the VAS scores for low back ache and right and left leg pain. However, the VAS values for left leg discomfort, right leg pain, and low back pain did not differ statistically substantially. For low back ache, the pre-surgery VAS was 7.46 ± 0.69 in both groups, and post-surgery, the scores were 2.7 ± 0.59 for TLIF and 2.9 ± 0.71 for PLF (p = 0.28). The variation in low back pain scores was -4.75 ± 1.04 for TLIF and -4.53 ± 1.07 for PLF (p = 0.42). Likewise, there were no appreciable variations in the group's improvements in leg pain. Multiple studies found that both procedures significantly improved pain and disability scores [1,16,17]. A study conducted by Fujimori et al. demonstrated that TLIF showed superior outcomes in reducing leg pain and restoring disk height compared to PLF [2].

According to a study conducted by Goh et al.), individuals with poor mental health at baseline who underwent TLIF saw notable reductions in pain and impairment that were on par with those who had normal mental health [18]. In our study, the SF-12 Health Survey showed a statistically important improvement in the TLIF unit as compared to the PLF unit in the PCS score. Before surgery, SF-12 PCS was 30.1 ± 1.7 for TLIF and 28.9 ± 4 for PLF (p = 0.53), and post-surgery, it was 42.4 ± 5.1 for TLIF and 37.9 ± 4.5 for PLF (p = 0.01). The change in SF-12 PCS scores was also greater in the TLIF group, with an improvement of 12.4 ± 4.1 compared to 9.6 ± 2.7 in the PLF unit (p = 0.07). The MCS ratings for the two groups did not differ substantially. These results indicate that TLIF may offer a greater improvement in physical function compared to PLF, although both groups had similar mental health outcomes.

In our study, radiographic parameters revealed notable differences between the two groups. Regarding slippage, the pre-surgery measurements were 5.0 ± 1.8 for TLIF and 5.5 ± 1.9 for PLF (p = 0.36). However, post-surgery, the TLIF group showed a greater reduction in slippage (2.1 ± 1.02) compared to the PLF group (3.1 ± 1.9), with a noteworthy p-value of 0.01. The change in slippage was -2.9 ± 0.98 for TLIF and -2.4 ± 0.84 for PLF (p = 0.03), indicating better control of vertebral slippage in the TLIF group. Few studies suggest that TLIF demonstrates superior radiographic outcomes, including better reduction of vertebral slippage, greater restoration of disc height, and improved local disc lordosis [2,9]. Open PLIF achieves higher slip reduction rates than minimally invasive TLIF [19].

Similarly, in the present study, the TLIF group demonstrated better outcomes in terms of local disc lordosis. The pre-surgery local disc lordosis for TLIF was 6.1 ± 2.5 compared to 4.7 ± 2.0 for PLF (p = 0.03). Post-surgery, the TLIF unit had a mean local disc lordosis of 7.4 ± 2.0, while the PLF group had a mean local disc lordosis of 5.5 ± 1.7 (p < 0.01). The TLIF group experienced a larger change in lordosis (1.3 ± 1.2) compared to the PLF group (0.79 ± 0.62); despite this, the change was not statistically significant (p = 0.06). Several studies show that TLIF demonstrated superior outcomes in restoring disc height and improving local disc lordosis compared to PLF [2,9,20].

In this study, in terms of anterior and posterior disc height, the TLIF group showed significantly greater improvements. The pre-surgery anterior disc height was 9.6 ± 1.8 for TLIF and 8.3 ± 1.4 for PLF (p < 0.01). Post-surgery, the TLIF group had an average anterior disc height of 11.3 ± 1.6, while the PLF group had an average anterior disc height of 9.3 ± 1.5 (p < 0.01). Research conducted by Kaliya-Perumal et al. reported that TLIF resulted in larger advancements in the anterior and the height of the posterior disc in connection with PLF [21].

In our study, the modification in anterior disc height was 1.7 ± 0.84 for TLIF and 1.0 ± 0.7 for PLF (p < 0.01). Similarly, for posterior disc height, the pre-surgery measurements were 6.1 ± 0.9 for TLIF and 5.2 ± 0.8 for PLF (p < 0.01), and post-surgery, they were 7.8 ± 0.53 for TLIF and 6.0 ± 0.7 for PLF (p < 0.01). The elevation of the height of the posterior disc was 1.7 ± 0.9 for TLIF and 0.8 ± 0.4 for PLF (p < 0.01), showing that TLIF resulted in better disc height restoration. A study by Hsieh et al. reported that anterior lumbar interbody fusion is better than TLIF in the restoration of foraminal height, angle of disc, and lumbar lordosis [22].

Limitation

The study's extremely small sample size may restrict how broadly the results may be applied to more significant, more varied populations. Furthermore, the study's brief follow-up period might have limited its ability to adequately capture the long-term effects and any side effects of TLIF and PLF operations. Selection bias may have been introduced by the lack of randomization between groups since factors not included in the study may have influenced the surgical approach chosen.

## Conclusions

This study found that both TLIF and PLF lead to significant improvements in clinical outcomes, but TLIF demonstrated superior results in terms of physical function, radiographic outcomes (such as reduction in slippage, improvement in disc height, and lordosis), and overall pain relief. Though the clinical outcome in the TLIF group was slightly better, it was not statistically significant. These results might imply that TLIF may be a more successful surgical technique in patients undergoing lumbar spine fusion, especially in terms of post-surgery recovery and long-term spinal alignment. Future studies should use bigger sample sizes and longer follow-up intervals to verify these results and evaluate the long-term advantages of TLIF over PLF.
